# Novel Genetic Variants of *PPARγ2* Promoter in Gestational Diabetes Mellitus and its Molecular Regulation in Adipogenesis

**DOI:** 10.3389/fendo.2020.499788

**Published:** 2021-01-22

**Authors:** Ling Wu, Yi Song, Yuan Zhang, Bo Liang, Yan Deng, Tao Tang, Yan Chou Ye, Hong Ying Hou, Chi Chiu Wang

**Affiliations:** ^1^Department of Obstetrics and Gynaecology, Prince of Wales Hospital, The Chinese University of Hong Kong, Hong Kong, Hong Kong; ^2^Department of Obstetrics and Gynecology, The Third Affiliated Hospital of Sun Yat-Sen University, Guangzhou, China; ^3^Development and Reproduction Laboratory, Li Ka Shing Institute of Health Sciences, The Chinese University of Hong Kong, Hong Kong, Hong Kong; ^4^School of Biomedical Sciences, The Chinese University of Hong Kong, Hong Kong, Hong Kong; ^5^Chinese University of Hong Kong-Sichuan University Joint Laboratory in Reproductive Medicine, The Chinese University of Hong Kong, Hong Kong, Hong Kong

**Keywords:** genetic variant, gestational diabetes, promoter, adipogenesis, molecular study

## Abstract

Peroxisome proliferator-activated receptor γ2 (*PPARγ2*) is a nuclear hormone receptor of ligand-dependent transcription factor with a key role in adipogenesis and insulin sensitization in diabetes mellitus. In this study, we investigated genetic variants in *PPARγ2* promoter, its association with gestational diabetes mellitus (GDM), and its molecular regulation. *PPARγ2* promoter and start codon (-2,091 to +82 bp) from 400 pregnancies with GDM and 400 gestational-age matched control pregnancies were sequenced. Association and linkage disequilibrium of the identified polymorphisms with GDM was determined. ChIP-seq, gene silencing, and dual-luciferase reporter assays were performed to confirm transcription factor binding sites and promoter activity of the variants. Transfection experiments were carried out to determine the effects of variants on gene expression and adipogenesis. Among 15 variants identified, 7 known variants were not significantly associated with the risk of GDM (odds ratio: 0.710–1.208, 95% confidence interval: 0.445–0.877 to 1.132–1.664, *P* > 0.05) while linkage disequilibrium was significant (D’ > 0.7, R^2^ > 0.9). However, T-A-A-T-G haplotype was not significantly associated with GDM (χ^2^ = 2.461, *P* = 0.117). Five rare variants and 3 novel variants (rs948820149, rs1553638909, and rs1553638903) were only found in GDM. Transcription factor glucocorticoid receptor β (GRβ) bound to -807A/C (rs948820149) and knockdown of GRβ suppressed *PPARγ2* promoter activity. This mutation significantly down-regulated *PPARγ2* expression and alleviated adipogenesis. In conclusion, a novel -807A/C in *PPARγ2* promoter was identified in Chinese women with GDM and the mutation affected GRβ binding and transcription of *PPARγ2* for adipogenesis.

## Introduction

Gestational diabetes mellitus (GDM) is defined as the first recognition of glucose intolerance during pregnancy. Interaction between genetic and environmental factors plays an important role in the development of GDM (1–3). Genome-wide association studies (GWAS) (4, 5) and meta-analyses (6–8) showed that GDM and Type 2 diabetes mellitus (T2DM) shared similar genetic background. Although genetic variants in Transcription Factor 7-like 2 (*TCF7L2*) (9, 10), Peroxisome Proliferator Activated Receptor *γ* (*PPARγ*) (11), Insulin Receptor Substrate 1 (*IRS1*), Melatonin Receptor 1B (*MTNR1B*) (12, 13), Glucokinase (*GCK*), Insulin Like Growth Factor 2 mRNA Binding Protein 2 (*IGF2BP2*), Potassium Voltage-Gated Channel Subfamily J Member 11 (*KCNJ11*), Potassium Voltage-Gated Channel Subfamily Q Member 1 (*KCNQ1*) (14, 15), and CDK5 Regulatory Subunit Associated Protein 1 Like 1 (*CDKAL1*) are involved in both GDM and T2DM risk, their effect sizes for the risk vary in different genes.

*PPARγ* is a nuclear hormone receptor which regulates cell growth, differentiation and metabolism in different organs and tissues (16). Compared with other *PPARγ* isforms, exon B is specific in isoform 2 (*PPARγ2*) which has extra 28 amino acids at NH2-terminal, leading to 5- to 10-fold ligand-independent activation. *PPARγ2* expression is mainly identified in adipocytes under physiological conditions (17). *PPARγ2* is specifically essential for effective adipogenesis (18), and *PPARγ2*-knockout obese POKO mice showed decrease of fat accumulation in adipocytes. PPARγ2 can prevent lipotoxicity by promoting the adipose tissue expansion and augmenting lipid-buffering capacity in peripheral organs (19). On the other hands, suppressing phosphorylation of serine 112 of *PPARγ2* in mice fed with high-fat diet (20), and human carrying mutation blocking phosphorylation of *PPARγ2* can enhance insulin sensitivity (21). In late pregnancy of *PPARγ2* knockout mice, *PPARγ2* is essential in promoting healthy adipose tissue expansion and immune and metabolic functionality during pregnancy, suggesting its association with the development of GDM (22).

A missense mutation of Pro12Ala in exon B (rs1801282) of *PPARγ2* gene is associated with low body mass index (BMI), insulin sensitivity and low risk of T2DM (23–25). In a large-scale study, 7% of elevated insulin sensitivity was observed in Swedish Ala allele male carriers with normal glucose tolerance. In a Finnish study, nondiabetic Ala allele carriers were associated with low BMI and decreased ligand-independent activity of *PPARγ2 (24)*. Both studies suggested the protective effects of Ala allele in diabetes at least in European populations (26, 27). However, genetic susceptibility of the variant may vary in different populations. Our previous meta-analysis and subgroup analysis showed that the rs1801282 variant was significantly associated with decreased risk of GDM only in Asian (4 studies, 1,197 cases versus 1,026 controls, odds ratio (OR) 0.72, 95% confidence interval (CI) 0.56-0.93), but not in Caucasian (6 studies, 1,732 cases versus 5,943 controls, OR 1.07, 95% CI 0.91–1.18) (5).

Exon B is near the start codon of *PPARγ2*, variants in promoter region may also affect the gene expression and its function. However, there is no data available in GDM. In this study, we investigated polymorphisms of *PPARγ2* promoter and exon B regions in Chinese pregnant women complicated with GDM. Novel genetic variants were identified only in GDM and its molecular regulation in gene expression and adipogenesis was studied.

## Research Design and Methods

### Subjects

Pregnant women at second trimester attended antenatal clinic of the Third Affiliated Hospital of Sun Yat-Sen University in Guangzhou between September 2013 and September 2015 for oral glucose tolerance test (OGTT) were included. The prevalence of GDM during the study period was 16.8%. The present study was approved by Ethics Committee of the Third Affiliated Hospital of Sun Yat-Sen University. All experimental procedures were performed in accordance with the relevant guidelines and regulations. Written informed consent was obtained from each patient to collect extra blood samples for genetic test. Diagnostic criteria of GDM was based on 75 g OGTT according to the International Association of the Diabetes and Pregnancy Study Groups (28). GDM were confirmed when blood glucose level was higher than 5.1, 10.0, 8.5 mmol/L at 0, 1, 2 h at any time point; controls were defined when the blood glucose levels were normal at all the time points. A total of 400 pregnant women diagnosed with GDM and 400 controls at 24–28 gestational weeks were included. The fasting total cholesterol, triglyceride, high-density lipoprotein, low-density lipoprotein, and HbA1C were also measured. Subjects with T1DM or T2DM, cardiovascular diseases (including hypertension and arteriosclerosis) and other metabolic disorders (including obesity BMI>30kg/m^2^ and polycystic ovary syndrome) were excluded. The whole blood samples collected during OGTT were stored in -80°C prior to analysis.

### Variant Identification and Genotyping

Promoter (chromosome 3:12351302∼12351501) and exon B regions (chromosome 3:12351502∼12351637) of *PPARγ2* were sequenced by Sanger sequencing. Genomic DNA was extracted from the whole blood sample by Magnetic Beads Automatic DNA Extraction Apparatus (HEASBioTech, Guangzhou, China). DNA quality was checked by NanoDrop 1000 Spectrophotometer (Thermo Fisher Scientific, Waltham, USA). DNA with A260/A280 ratio within 1.6–1.8 was regarded as high quality for sequencing. Sequencing primers were designed by Primer3 version 4.0.0. Four pairs of primers covering the whole promoter and exon B regions were used (**Supplementary Table 1**). PCR was performed by PCR kit (HEASBioTech, Guangzhou, China) according to the manufacturer’s instructions. Single nucleotide polymorphisms (SNPs) were identified according to the reference sequence of *PPARγ2* in Ensemble Human Genome Assembly (GRCh38.p12). Minor allele frequency (MAF) was calculated as the frequency of less frequent allele in a given locus and a given population. Known variant was defined as variant that has been previously identified and reported in public database, such as dbSNP. Novel variant was defined as a newly discovered variant and has never been reported anywhere. Common variant was defined as the variant with minor allele frequency (MAF) >5%; rare variant as the known variant with minor allele frequency (MAF) <0.5% (29). Pooled odds ratios (ORs) and 95% confidence intervals (CIs) were calculated. Allele model was used to compare the risk allele with wild type allele. Hardy Weinberg Equilibrium (HWE) was defined as allele and genotype frequencies in a population remained constant from generation to generation in the absence of other evolutionary influences (30). Statistical significant level was set at *P <*0.05. Linkage disequilibrium was carried by Haploview software, D’ > 0.7, R^2^ > 0.33 referred as strong linkage disequilibrium. Genetic analysis was carried out by Sequence Scanner Software v1.0 (Applera Corp. USA).

### Cell Lines

Cell lines were used for molecular and functional studies of the identified variants. Human Embryonic Kidney cells (HEK293) were purchased from American Type Culture Collection (Manassas, USA) and cultured in DMEM (Thermo Fisher Scientific) supplemented with 10% fetal bovine serum (FBS; Thermo Fisher Scientific) and 1% penicillin and streptomycin (Sigma-Aldrich). Human pre-adipocytes-subcutaneous (HPA-s) cells were purchased from ScienCell (Carlsbad, USA), cultured in Pre-adipocyte Medium supplemented with 10% FBS and 1% penicillin and streptomycin, and differentiated into mature adipocytes in Pre-adipocyte Differentiation Medium supplemented with 10% FBS and 1% PS according to previous report (31). Expression of *PPARγ2* was detected in both cultivated HEK293 cells and differentiated HPA-s cells. HEK293 cells were used for transcription factor siRNA silencing and luciferase reporter assays, and HPA-s cells were used for chromatin immunoprecipitation (ChIP)-PCR and transfection and functional studies.

### Transcription Factors and Silencing

To study transcription factors of the variants, transcription factor binding sites were predicted by PROMO 3.0.2 (32). To confirm the binding sites, ChIP-PCR was employed. HPA-s cells after 7 days of differentiation were cross-linked with 1% formaldehyde for 20 min, and glycine for 5 min. The cells were then washed with ice-cold phosphate buffered saline (PBS), and centrifuged at 1,000 g for 5 min. The cells were lysed in 1 ml lysis buffer, and sonicated at power 200 W at 55 s shock with 55 s interval for 15 min. 100 μl cell lysates were incubated as input with either anti-IgG antibody (Millipore, Burlington, USA) or anti-GRβ antibody (Abcam, Cambridge, UK). Immunoprecipitation was conducted with 0.9 ml IP Dilution Buffer, 70 μl Protein A beads, rotated for 60 min at 4°C, and centrifuged at 5,000 rpm for 2min. The target DNA was eluted with 200 and 300 μl Elusion Buffer twice, respectively. Reverse cross-link was performed with 20 μl 5 M NaCl overnight at 65°C, and then 10 μl 0.5 M EDTA, 20 μl 1 M Tris-HCl (pH 6.5), and 1 μl protein K (20mg/ml) at 45°C for 1.5 h. The DNA was purified by chloroform and ethanol. The purified DNA was subjected to the Sanger sequencing. The primer sequences are shown in **Supplementary Table 1**.

To silence the specific transcription factors of the variants, small interfering RNAs (siRNAs) targeting the transcription factors were purchased from RiboBio (Guangzhou, China). The reference sequences were obtained from genOFF™ siRNA library, and sequences of the siRNAs are shown in **Supplementary Table 2**. SiRNAs were transfected into HEK293 cells with Lipofectamine 2000 (Invitrogen, Carlsbad, USA) according to the manufacturer’s protocol (33). Transfection efficiency was confirmed by using Green Fluorescent Protein (GFP; RiboBio) as a reporter. Transfection efficiency was presented as percentage of number of cells stained with fluorescent positive stained cells over the total number of cells per field. GFP The transfected HEK293 cells up to 95% observed under electronic microscope were collected after 24 h for gene expression studies. Total RNA of the treated cells was extracted by RNA Extraction Kit (QIAGEN, Hilden, Germany). Quality of RNA was checked by NanoDrop 1000 Spectrophotometers (Thermo Fisher Scientific). RNA with A260/A280 ratio within 1.8–2.1 was regarded as high quality. RNA samples were then reverse transcribed to cDNA by using 1^st^ and 2^nd^ Strand cDNA Kit (TaKaRa, Tokyo, Japan). *PPARγ2* expression were quantified using Taqman Primers (Thermo Fisher Scientific) by a PCR system (HEASBioTech., Guangzhou, China) using SYBR Green Real-Time PCR Master Mixes (Takara). The relative mRNA expression of *PPARγ2* was normalized by *GAPDH* and calculated using the 2^-ΔΔCt^ method.

### Luciferase Reporter Assays

Firefly luciferase vector PGL3 (Sangon Biotech, Shanghai, China) was used to examine promoter activity of the variants. The putative promoter region with DNA fragment of wild type or mutant allele of ~982 bp length (from 100 bp upstream of the target allele to 100 bp downstream of transcription start site) was inserted to upstream of luciferin gene. Human NR3C1 CDS with 2,369 bp was inserted in pcDNA3.1 [EcoRI/XhoI] to over-express *PPARγ*. GRβ vector NC and PGL3 with *PPARγ*-Promoter of the wild type or mutant allele were co-transfected into HEK293 cells at 80% confluent by Lipofectamine 2000 reagent (Invitrogen). At 72 h after transfection, dual-luciferase reporter activity was determined by adding Luciferase Assay Buffer II and Stop & Glo^®^ Reagent using Dual Luciferase Reporter Assay kits (Promega, Madison, USA) according to manufacturer’s instruction. The firefly luciferase activity and Renilla luciferase activity were measured in EnSpire™ ultra-sensitive luminescence reader (PerkinElmer, Waltham, USA).

### Transfection and Functional Assays

Lentivirus pLVX-PGK-Puro expression vector with either wild type allele or risk allele and empty vector with puromycin resistance gene were provided by Biowit Biotech (Hefei, China). The putative promoter region with DNA fragment of wild type allele or risk allele and the whole CCDS of *PPARγ2* were inserted to the upstream of PGK promoter. HPA-s cells at 70–80% confluence in 96-well plate were transfected with the pLVX-PGK-Puro vector at 1 to 100 multiplicity of infection (MOI; 1×10^8^ TU/ml), and puromycin was used for transductant selection (34).

### Oil Red Staining of HPA-s Cells

After 3 days of puromycin selection, HPA-s cells were evaluated by Oil red staining. Briefly, the cells were washed with ice-cold phosphate buffered saline (PBS) for 3 times and fixed with 10% formalin at room temperature for 30 min. After that, the cells were rinsed with 60% isopropanol and were then stained with Oil Red O solution (Sigma-Aldrich) for 30 min followed by washing with 60% isopropanol. The stained cells were then imaged under a light microscope.

### Statistical Analysis

Data analysis was performed using the SPSS 27.0 (IBM, Armond, USA) and GraphPad Prism Software Version 5.0 (GraphPad Software, La Jolla, USA). The continuous data were presented as mean ± standard deviation, and significant differences between different groups were analyzed by unpaired Student’s t-test or one-way ANOVA followed by the Bonferroni’s post-hoc test, as appropriate. For the categorical data, the significant difference was analyzed using Chi-square test. Pooled odds ratios (ORs) and 95% confidence intervals (CIs) were calculated. Statistical significant level is set at *P <*0.05. Linkage disequilibrium was carried out by Haploview software, D’ > 0.7 and R^2^ > 0.33 were referred to strong linkage disequilibrium. Genetic analysis was conducted by Sequence Scanner Software v1.0 (Applera Corp.).

## Results

### Demographic Characteristics

In control group, 5 cases were excluded due to twin pregnancy, and duplicated and invalid samples. In GDM group, 2 cases were excluded due to the diagnosis of overt T1DM after delivery. Maternal age, pre-pregnancy BMI, glucose levels, fasting cholesterol and triglyceride levels were significantly higher and high-density lipoprotein level was significantly lower in GDM group than that in the control group (**Table 1**). The rate of advanced maternal age, family history of DM, previous history of GDM, and Caesarean section were significantly higher in GDM group than that in control group (**Table 1**). Maternal complications (postpartum hemorrhage, polyhydramnios, and shoulder dystocia) and neonatal complications (hypoglycemia and macrosomia) were not significantly different between groups (**Table 1**). The univariate and multivariate logistic regression showed increased maternal age, family history of DM and previous history of GDM were significantly associated with the GDM in the current pregnancy (**Table 2**).

**Table 1:** Demographic and clinical characteristics.

Demographic and Clinical Profiles	Controls (n = 395)	GDM (n = 398)	P value
Maternal Age (yr)	29.2 ± 4.3	31.4 ± 4.4	<0.01
Pre-pregnancy BMI (kg/m^2^)	20.4 ± 2.7	21.4 ± 3.5	<0.01
Advanced maternal age (≥ 35 yr)	47 (11.9%)	84 (21.1%)	<0.01
Previous history of miscarriage	45 (11.4%)	64 (16.1%)	0.10
Family history of DM and previous GDM	24 (6.1%)	62 (15.6%)	<0.01
Premature delivery (<37 wk)	16 (5.4%)	24 (8.1%)	0.13
Low birth weight	20 (5.1%)	32 (8.0%)	0.16
OGTT_0 h (mmol/L)	4.2 ± 0.4	4.6 ± 0.8	<0.01
OGTT_1 h (mmol/L)	6.1 ± 3.0	9.0 ± 3.1	<0.01
OGTT_2 h (mmol/L)	5.5 ± 2.6	8.2 ± 2.9	<0.01
Cholesterol (mmol/L)	5.0 ± 1.2	5.2 ± 1.2	0.02
Triglyceride (mmol/L)	1.5 ± 0.8	2.0 ± 1.0	<0.01
High-density lipoprotein (mmol/L)	1.8 ± 0.4	1.7 ± 0.4	0.01
Low-density lipoprotein (mmol/L)	2.6 ± 0.8	2.8 ± 0.9	0.06
Pregnancy outcomes			
Gestational weeks at delivery (wk)	38.7 ± 1.5	38.4 ± 5.4	0.37
Cesarean section	121 (30.6%)	163 (41.0%)	<0.01
Postpartum hemorrhage	8 (2.0%)	4 (1.0%)	0.25
Polyhydramnios	10 (2.5%)	6 (1.5%)	0.31
Shoulder dystocia	0 (0%)	0 (0%)	0.99
Neonatal outcomes of complications			
Hypoglycemia	2 (0.5%)	3 (0.8%)	0.66
Macrosomia	14 (3.5%)	10 (2.5%)	0.99

BMI, body mass index; DM, diabetes mellitus; GDM, gestational diabetes mellitus; wk, week; yr, years old.

**Table 2:** Confounding factors and risk factors for GDM with univariable and multivariable logistic regression.

	Univariable			Multivariable		
	OR	95% CI	P values	OR	95% CI	P values
Maternal Age (yr)	1.130	1.092–1.169	0.000	1.135	1.068–1.206	<0.01
Multipara	0.687	0.517–0.912	0.009	0.718	0.430–1.199	0.21
Previous history of miscarriage	0.649	0.433–0.973	0.036	1.752	0.816–3.759	0.15
Premature delivery (<37wk)	0.976	0.924–1.030	0.379	1.028	0.982–1.076	0.24
Cesarean section	0.641	0.478–0.859	0.003	1.149	0.686–1.923	0.6
Low birth weight	1.000	1.000–1.000	0.602	1	0.999–1.000	0.28
Family History of DM and previous GDM	0.287	0.162–0.506	0.000	2.804	1.256–6.260	0.01
Pre-pregnancy BMI (kg/m2)	1.174	1.111–1.240	0.000	0.795	0.422–1.497	0.8
Cholesterol (mmol/L)	1.396	1.177–1.654	0.000	1.425	0.821–2.473	0.21
Triglyceride (mmol/L)	2.236	1.728–2.893	0.000	1.478	0.995–2.196	0.05
High-density lipoprotein (mmol/L)	0.525	0.318–0.867	0.012	0.61	0.237–1.575	0.31
Low-density lipoprotein (mmol/L)	1.399	1.103–1.774	0.006	0.741	0.395–1.391	0.35

BMI, body mass index; DM, diabetes mellitus; GDM, gestational diabetes mellitus; wk, week; yr, years old.

### Genotyping, Allele, and Linkage Analysis

**Figure 1:**
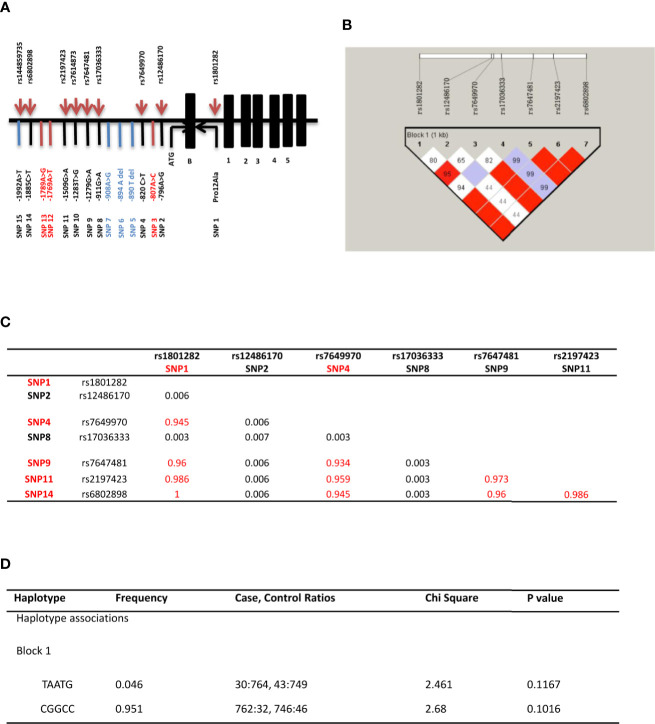
*PPARγ2* gene associated study. **(A)** Distribution of the 15 SNPs across the promoter region and the start codon region of *PPARγ2* gene. Positions are relative to the translation initiation site, ATG; 15 variants identified in the *PPARγ2* promoter region and start codon region (—1,997 to +82 bp); Common variants (SNP1 2 4 8 9 11 14); Rare variants (SNP 3 5 6 7 10 12 13 15); and Novel SNPs (SNP 3 12 13). **(B)** Linkage estimates (r^2^) * between *PPARγ2* gene variants. r^2^ more than 0.33 means strong LD. **(C)** Linkage disequilibrium (LD) blocks detected by Haploview software. rs numbers for each polymorphism are individualized. Numbers in boxes indicate the LD (based on D’) for each pair of variants; SNPs with red matrices are strong LD. **(D)** Common haplotype frequencies and association analyses.

A total of 15 variants in the promoter region and start codon of *PPARγ2* gene were found in GDM and control groups (**Table 3** and **Figure 1A**). Seven out of 9 known variants were common variants (rs1801282, rs12486170, rs7649970, rs17036333, rs7647481, rs2197423, and rs6802898) and three were rare variants (rs17036333, rs7614873 and rs144859735). Three out of 6 unknown variants (-890T/-, -894A/- or A/G and -908A/- or A/G) were novel variants, which were identified in both groups, while the other three novel variants (-807A/C, -1769A/T, and -1789A/G) were only found in the GDM group. In allele analysis, none of the variants was significantly associated with the risk of GDM (**Supplementary Table 3**) and overweight (**Supplementary Table 4** and **5**).

**Table 3:** Variants of the PPARγ2 promoter and start codon region.

SNP No.	Chr.	Chromosome Position	Position (relative to ATG)	Assession number**	Mutation Type	Wild allele/Minor allele	1000G MAF	Control	GDM	OR (95% CI)(Allele model)	P value	HWEP value
1	3	12351626	Pro12ala	rs1801282	common	C/G	G = 0.0703	44(11.1%)	32(8.0%)	0.710 (0.445, 1.132)	0.15	0.24
2	3	12350797	-796	rs12486170	common	A/G	G = 0.1687	72(18.2%)	86(21.6%)	1.208 (0.887, 1.664)	0.247	0.63
3	3	12350786	-807	rs948820149	novel	A/C	NA	0	1(0.3%)	NA	NA	NA
4	3	12350773	-820	rs7649970	common	C/T	T = 0.1198	44(11.1%)	32(8.0%)	0.710 (0.445, 1.132)	0.15	0.24
5	3	12350703	-890	NA	rare	T/-	NA	1(0.3%)	0	NA	NA	NA
6	3	12350699	-894	NA	rare	A/-or A/G	NA	1(0.3%)	1(0.3%)	NA	NA	NA
7	3	12350685	-908	NA	rare	A/-or A/G	NA	2(0.5%)	1(0.3%)	NA	NA	NA
8	3	12350682	-911	rs17036333	rare	G/A	A = 0.0086	40(10.1%)	41(10.3%)	0.963 (0.619, 1.499)	0.868	0.91
9	3	12350314	-1279	rs7647481	common	G/A	A = 0.1198	45(11.4%)	33(8.3%)	0.716 (0.452, 1.135)	0.155	0.23
10	3	12350310	-1283	rs7614873	rare	T/G	G = 0.0405	1(0.3%)	0	NA	NA	NA
11	3	12350084	-1509	rs2197423	common	G/A	A = 0.1198	44(11.1%)	33(8.3%)	0.733 (0.462, 1.164)	0.189	0.24
12	3	12349824	-1769	rs1553638909	novel	A/T	NA	0	1(0.3%)	NA	NA	NA
13	3	12349804	-1789	rs1553638903	novel	A/G	NA	0	1(0.3%)	NA	NA	NA
14	3	12349708	-1885	rs6802898	common	C/T	T = 0.2576	44(11.1%)	33(8.3%)	0.733 (0.462, 1.164)	0.189	0.24
15	3	12349601	-1992	rs144859735	rare	A/T	T = 0.0016	0	1(0.3%)	NA	NA	NA

Pro12ala locates in codon region; SNP5～7 are not reported, so no rs numbers but were found all in control.

New reference numbers for novel SNPs come from NCBI (National Center for Biotechnology Information).

Amongst seven common variants, five variants (rs1801282, rs7649970, rs7647481, rs2197423 and rs6802898) showed significant linkage disequilibrium (D’ > 0.7, R^2^ > 0.9; **Figures 1B, C**). However, the T-A-A-T-G haplotype was not significantly associated with the risk of GDM (χ^2^ = 2.461, *P* = 0.117; **Figure 1D**). No statistical significant association between T-A-A-T-G and C-G-G-C-C haplotypes were found with any demographic and clinical characteristics (**Supplementary Table 6**).

### Transcription Factors and Binding of the Novel Variants

**Figure 2:**
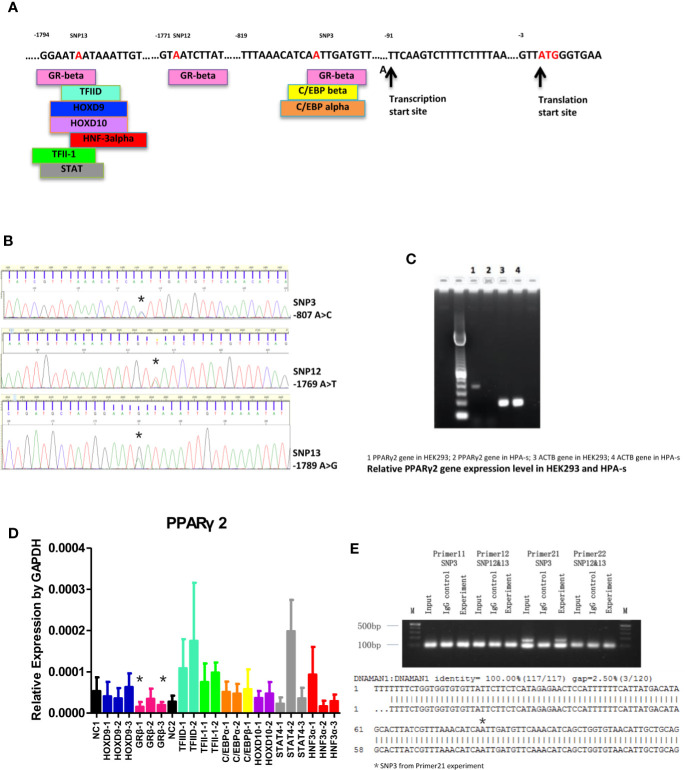
*PPARγ2* gene molecular study. **(A)** Transcription factors binding sites of TFBS and association with the locations of novel SNPs (red). **(B)** Sequencing chromatograms of 3 novel SNPs (*location of the nucleotide changes; -807A/C, -1769A/T, -1789A/G). **(C)** Gel electrophoresis of PPARγ2 gene expression in HEK293 and HPA-s cells. **(D)** The effects of different siRNAs on *PPARγ2* expression level. *GRβ-1 and GRβ-3 significantly knocked down 67 and of 69% *PPARγ2* expression levels. N = 3 for each group. **(E)** Upper panel, Gel electrophoresis of the novels SNPs binding to GRβ antibody by ChIP-PCR. Primers 11 and 22 designed for -1769A>T and -1789A>G; Primers 11 and 21 designed for -807A>C. Lower panel, Sequence alignment between *PPARγ2* promoter fragment, *-807A/C detected by the primer 21. N = 3 for each group.

Nine transcription factors, including homeobox D9 (HOXD9), GRβ, TATA binding protein-1 (TFIID-1), TATA binding protein-2 (TFIID-2), CCAAT enhancer binding protein alpha (C/EBP alpha), CCAAT enhancer binding protein beta (C/EBP beta), general transcription factor II I (TFII-1), homeobox D10 (HOXD10), signal transducer and activator of transcription 4 (STAT4) and forkhead box A1 (HNF3 alpha), were predicted to bind to the mutant allele (**Figures 2A, B**). As shown in **Figure 2C**, PPARγ2 gene could be detected in HEK293 cells, but could hardly be detected in undifferentiated HPA-s cells. However, only GRβ siRNAs significantly suppressed *PPARγ2* expression in HEK293 cells (**Figure 2D**), suggesting GRβ is a potential transcription factor of *PPARγ2*. Immunoprecipitation showed that GRβ bound to -807A/C, but not -1769A/T or -1789A/G (**Figure 2E**, see the original gel results in **Supplementary Figure 1**). Taken together, the results suggested that GRβ was a transfection factor specifically bind to -807A/C, which regulated the *PPARγ2* expression.

### Promoter Activity of -807A/C and Functional Studies

**Figure 3:**
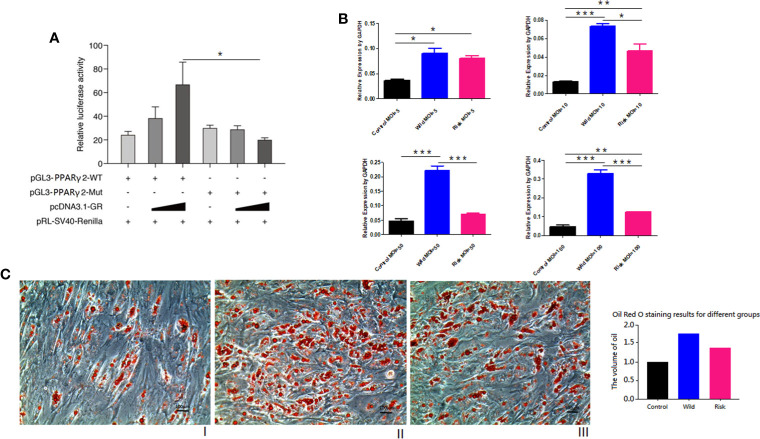
*PPARγ2* gene functional study. **(A)** GR-beta NC/over-expressed vector & PGL3-*PPARγ*-Promoter-MT/Mut co-transfected by Lipofectamine 2000 in HEK293. Relative luciferase activity (normalized to Renilla luciferase). GRβ over-expressed vector with high concentration and PGL3-*PPARγ*-Promoter-MT/Mut, SNP3-mut significantly down-regulated promoter activity (P = 0.0138); N = 3 for each group. **(B)** pLVX-PGK-Puro vector were transfected in HPA-s. Empty vector vs. SNP3 wild allele vector vs. SNP3 mutant vector shows SNP3 mutant significantly down-regulated *PPARγ2* gene expression level (*P* < 0.0001). MOI = 5 (*P* = 0.3818); MOI = 10 (*P* = 0.0246); MOI = 50 (*P* = 0.0178); MOI = 100 (*P* = 0.0007). **(C)** Oil red staining for HPA-s after differentiation at 7^th^ day. (I) Normal cells, (II) HPA-s cells with wild type allele transfected with pLVX-PGK-Puro vector (MO I= 50) (III) HPA-s cells with risk allele transfected with pLVX-PGK-Puro vector (MOI = 50). N = 3 for each group. The -807A/C variant limited PPARγ2 expression and alleviated accumulation of oil droplets. *0.01 < P value < 0.05; **0.001 < P value < 0.01; ***0.0001 < P value < 0.001.

PPARγ2 expression levels in HPA-s cells were gradually increased during differentiation. (**Supplementary Figure 2**). Transfection results showed that SNP3-mutant significantly down-regulated the promoter activity in a concentration-dependent manner (*P* = 0.0138, **Figure 3A**), suggesting the reduced transcription activity of *PPARγ2* promoter carrying the variant -807A/C. Transfection of lentivirus pLVX-PGK-Puro expression vector with the mutant -807A/C variant in HPA-s cells significantly down-regulated the *PPARγ2* gene expression at MOI = 10 (*P* = 0.0246), MOI = 50 (*P* = 0.0178) and MOI = 100 (*P* = 0.0007) (**Figure 3B**), and also limited accumulation of oil droplets in the HPA-s cells after differentiation (**Figure 3C**). Taken together, the results suggested that the -807A/C variant limited *PPARγ2* expression and alleviated adipogenesis.

## Discussion

For the first time our study showed that there was no significant association of *PPARγ2* variants in promoter and exon B with susceptibility of GDM in the Chinese women population. However, we identified novel variant important for GRβ binding and its regulatory potential in adipogenesis. *PPARγ*-RXR heterodimer promotes recruitment of co-activator complex and regulates different physiological processes, including glucose homeostasis in white adipocytes for glucose metabolism and lipid metabolism in both white and brown adipocytes and dendritic cells (35). Mutations in *PPARγ* gene retain the ability to heterodimerize with RXR but fail to bind to ligands for recruiting co-repressor complex, which may subsequently lead to the silence of gene transcription.

In our study, no association between the common variants in promoter and exon B of *PPARγ2* and GDM risk was found. The negative results may be due to the sample size in current study not large enough to detect the variants at a low frequency. However, five variants, including rs1801282, found to be significant linkage disequilibrium in our case-control cohort. The rs1801282 is located in codon 12 of exon B and was first discovered in 1997 (36). Exon B is the first coding region of *PPARγ2*, and the promoter region and exon B are just next to each other. Mutation in promoter region will likely affect the exon B and initiation of the transcription. The minor allele frequencies in exon B vary in different populations, such as 12% in Caucasian populations; 10% in Native American populations; 4% in Japanese; 3% in African-American and 1% in Chinese. In Caucasian populations, the highest frequency was found up to 25% (37). Inconsistent results were reported regarding the association between rs1801282 and GDM risk. Our previous meta-analysis and subgroup analysis reported that this polymorphism was not associated with GDM risk in pooled populations, but significantly associated with the decreased risk of GDM in Asian population (7). From Exome Aggregation Consortium database (ExAC, sample size 121,412), the overall risk allele frequency of the rs1801282 polymorphism in general Asian population is 0.1199. However, in our meta-analysis, the overall risk allele frequencies of rs1801282 in GDM Asian population (sample size 1,259) and other populations (sample size 1,285) were 0.0434 and 0.1118, respectively. Here in our genetic study of South Chinese woman, the overall risk allele frequencies of rs1801282 in GDM and control Asian population are 0.04 and 0.06, respectively. The prevalence of rs1801282 is lower in control group than that in general population. Reason of the ethical differences is unknown, the prevalence of rs1801282 may be different in the South and North Chinese population (38–42), but the HWE of rs1801282 in control group was not significant, confirming no sampling bias.

Molecular study of novel variants showed that C allele of -807A/C significantly up-regulated promoter activity of *PPARγ2* comparing to A allele. Although T allele of -1769A/T up-regulated promoter activity of *PPARγ2* comparing to A allele but GRβ did not bind to -1769A/T or -1789A/G. On the other hand, C allele of -1789A/C down-regulated promoter activity of *PPARγ2* comparing to A allele and GRβ siRNA significantly down-regulated the activity. Interestingly, the binding site of -1789A/C cannot be confirmed by ChIP-PCR. GRβ could be regulated by other genes and could indirectly regulate *PPARγ2* transcription. GR is a member of the nuclear hormone receptor superfamily and a ligand-dependent transcriptional activator. Glucocorticoids regulate numerous physiological processes by binding to the GR (43). Synthetic glucocorticoids are widely used for inflammatory diseases, autoimmune disorders and cancers treatment (44). GR has 2 isoforms, GRα and GRβ, which are generated by different splicing sites from the same transcripts of GR gene (45). GRα plays an important role in the physiological and pharmacological functions of glucocorticoids, while GRβ suppresses GRα expression through its altered ligand-binding domain (46). Studies reported that GRβ was associated with glucocorticoids insensitivity, asthma, chronic obstructive pulmonary disease (47, 48), allergic rhinitis (49), human bladder cancer (50), T2DM (51), adipogenesis, and obesity (52). Isoforms of GR as chaperone proteins induced glucocorticoids resistance and promoted lipogenesis by glucocorticoid activation of the mineralocorticoid receptor (52). On the other hand, dexamethasone (DEX) is a synthetic ligand for GR, which stimulates adipogenesis. DEX-bound GR accelerates adipogenesis by promoting gene expression of adipogenic transcription factors CCAAT/enhancer-binding protein alpha (C/EBP), C/EBP, C/EBP, KLF5, KLF9, and also *PPAR* in the early phase of differentiation (53). In our study, the expression level of GRβ was elevated during the HPA-s cell differentiation, which was consistent with the elevated *PPARγ* expression.

Several limitations should be addressed in the present study. In our case control study, we found individual with multiple variants in *PPARγ2*. In linkage analysis, rs1801282, rs7649970, rs7647481, rs2197423, and rs6802898 shown significant correlation, but no significant difference was found between control and case groups. And also, no significant correlation regarding the novel SNPs was found in our small study population. On the other hand, based on our previous meta-analysis of genetic variants in GDM, *PPARγ2* directly linked with *TNF* and *MTNR1B* and indirectly with *IRS1* and *TCF7L2*. However, there is also no single study determined the risk of GDM in combination of the variants. Up to date, there is no available data regarding the association between rs948820149 in the *PPARγ2* promoter region and T2DM or obesity. Since the identified genetic variant is novel, it had never been identified in other diabetes. Small study population in other studies may be the reason. Further large-scale study is necessary to determine the variant in promoter and exon B with susceptibility of T2DM and obesity.

In conclusion, the common genetic variants in promoter and start codon region of *PPARγ2* were not significantly associated with the risk of GDM. The common variants (rs1801282, rs7649970, rs7647481, rs2197423, and rs6802898) have significant linkage disequilibrium but not significantly associated with the risk of GDM. Importantly, 3 novel SNPs (-807A/C, -1769A/T, and -1789A/C) were detected only in GDM group. While -807A/C variant (rs948820149) bound to the transcription factor GRβ and regulated the transcription of *PPARγ2* in adipogenesis. Identification of genetic variants may allow early intervention in pre-conception or early pregnancy period in order to prevent maternal and pre-/post-natal complications.

## Data Availability Statement

All datasets generated/analyzed for this study are included in the article/**Supplementary Material**.

## Ethics Statement

The studies involving human participants were reviewed and approved by The Third Affiliated Hospital of Sun Yat-Sen University. The patients/participants provided their written informed consent to participate in this study. Written informed consent was obtained from the individual(s) for the publication of any potentially identifiable images or data included in this article.

## Author Contributions

LW, CCW, and TT designed the research studies. YZ, YCY, and HYH provided blood samples. LW, YS, BL, and YD conducted experiments. LW and YS acquired data and analyzed data. LW and CCW wrote the manuscript. CCW revised the manuscript. All authors contributed to the article and approved the submitted version.

## Conflict of Interest

The authors declare that the research was conducted in the absence of any commercial or financial relationships that could be construed as a potential conflict of interest.
