# Supplementary material for: Novel Genetic Variants of PPARγ2 Promoter in Gestational Diabetes Mellitus and its Molecular Regulation in Adipogenesis
Source: Front Endocrinol (Lausanne). 2021 Jan 22;11:499788. doi: 10.3389/fendo.2020.499788 (PMC7862745; doi:10.3389/fendo.2020.499788)
Supplement: Supplementary file 1 [file DataSheet_1.docx]

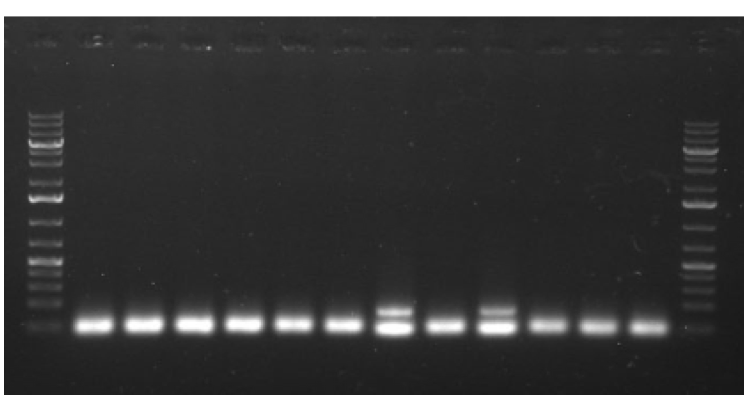


**Supplementary figure 1: original gel results.**

| **D0** | **D1** | **D2** | **D3** | **D4** | **D5** | **D6** | **D7** |
| --- | --- | --- | --- | --- | --- | --- | --- |
| 23.5593 | 23.3030 | 23.0529 | 23.5376 | 24.0883 | 23.9915 | 23.8976 | 23.2418 |
| 23.4388 | 23.3036 | 23.1090 | 23.5345 | 24.1405 | 23.9365 | 23.9914 | 23.2414 |
| 23.4585 | 23.3012 | 23.3100 | 23.5673 | 24.0502 | 23.9866 | 23.9945 | 23.1298 |

**Supplementary figure 2: PPARγ2 expression level in HPA-s cell before and after differentiation and Ct value of GAPDH from D0-D7**

**Supplementary Table 1** Primer sequences for promoter region and start codon of PPARγ2 gene and Primer sequences for ChIP-PCR

| **Primer pair** | **Code** | **Sequence （5’-3’）** | **Length** |
| --- | --- | --- | --- |
| **1*** | **1F** | AATCCACATTGTATAACTGTTTTGTT | 26 |
|  | **1R** | CCTTTCCTCCCCATGCTT | 18 |
| **2*** | **2F** | GCATCTGTGTGTTGCCAAAG | 20 |
|  | **2R** | TGAGAAGAATAACACACCACCA | 22 |
| **3*** | **3F** | GCCAGCTTTTTCCTGATTACA | 21 |
|  | **3R** | CTGCTTTGGCAAGACTTGGT | 20 |
| **4*** | **4F** | GGATATTGAACAGTCTCTGCTCTG | 24 |
|  | **4R** | CAAACACAACCTGGAAGACAAA | 22 |
| **5#** | **11F** | CTGGTGGTGTGTTATTCTTCTCA | 23 |
|  | **11R** | GCAACTGCAGCAATGTTACAC | 21 |
| **6#** | **21F** | TGGTGGTGTGTTATTCTCATAG | 22 |
|  | **21R** | CAACTGCAGCAATGTTACACC | 21 |
| **7##** | **12F** | TGTCAGAATGTGGTGGGTGC | 20 |
|  | **12R** | AGTTCCCATACTGTGCTTTGCTT | 23 |
| **8##** | **22F** | TCAGAATGTGGTGGGTGCTA | 20 |
|  | **22R** | TCAGAATGTGGTGGGTGCTA | 20 |

***** Primer sequences for promoter region and start codon of PPARγ2 gene

# Primer sequences for SNP3 for ChIP-PCR

## Primer sequences for SNP12 and SNP13 for ChIP-PCR

**Supplementary Table 2** Transcription factors siRNA sequences from genOFF^TM^ siRNA library

| Transcription factor | siRNA sequences (5’-3’) |
| --- | --- |
| HOXD9-1 | GAGTTCGCCTCGTGTAGTT |
| HOXD9-2 | ACTCGTTCCTGCAGGAGAA |
| HOXD9-3 | ACTCGCTTATAGGCCATGA |
| GR-beta-1 | GGATCATGACTACGCTCAA |
| GR-beta-2 | ACAGCACAATTACCTATGT |
| GR-beta-3 | CAGCATGCCGCTATCGAAA |
| TFIID-1 | GGTTTAATCTACAGAATGA |
| TFIID-2 | GCGGTAATCATGAGGATAA |
| C/EBP-alpha-1 | ACGAGACGTCCATCGACAT |
| C/EBP-alpha-2 | CGGTGGACAAGAACAGCAA |
| C/EBP-beta | CCATGGAAGTGGCCAACTT |
| TFII-1-1 | CGAGAACTATGATCTTGCA |
| TFII-1-2 | GTCGTGTGATGGTAACAGA |
| HOXD10-1 | GAACAGATCTTGTCGAATA |
| HOXD10-2 | CTTGCTCCTTCACCACCAA |
| STAT4-1 | GCCTGACCATAGATTTGGA |
| STAT4-2 | AACGGCTGTTGCTAAAGGA |
| STAT4-3 | TGGTCGTGGTCTTAACTCA |
| HNF-3 alpha-1 | CACTGCAATACTCGCCTTA |
| HNF-3 alpha-2 | AGACGTTCAAGCGCAGCTA |
| HNF-3 alpha-3 | TCCCGGTCAGCAACATGAA |

**Supplementary Table 3** Genotype and allele frequencies of the 7 common variants in the PPARγ2 gene

| Variants and genotypes | GDM | Control | OR, 95% CI  (#Dominant Model) | P value | OR, 95% CI  (*^¶^*Allele Model) | P value |
| --- | --- | --- | --- | --- | --- | --- |
| SNP1 (rs1801282) | | | | | | |
| Pro/Pro | 366 (92.0%) | 351 (88.9%) | 0.697 (0.432, 1.125) | 0.14 | 0.710 (0.445, 1.132) | 0.15 |
| Pro/Ala | 32 (8.0%) | 44 (11.1%) |  |  |  |  |
| Ala/Ala | 0 (0%) | 0 (0%) |  |  |  |  |
| *MAF (Ala) | 0.04 | 0.06 |  |  |  |  |
| SNP2 (rs12486170) | | | | | | |
| AA | 309 (77.6%) | 320 (81.0%) | 1.229 (0.871, 1.735) | 0.241 | 1.208 (0.877, 1.664) | 0.247 |
| GA | 86 (21.6%) | 72 (18.2%) |  |  |  |  |
| GG | 3 (0.8%) | 3 (0.8%) |  |  |  |  |
| MAF (G) | 0.12 | 0.10 |  |  |  |  |
| SNP4 (rs7649970) | | | | | | |
| CC | 366 (92.0%) | 351 (88.9%) | 0.697 (0.432, 1.125) | 0.14 | 0.710 (0.445, 1.132) | 0.15 |
| CT | 32 (8.0%) | 44 (11.1%) |  |  |  |  |
| TT | 0 (0%) | 0 (0%) |  |  |  |  |
| MAF (T) | 0.04 | 0.06 |  |  |  |  |
| SNP8 (rs17036333) | | | | | | |
| GG | 357 (89.7%) | 355 (89.6%) | 0.986 (0.624, 1.558) | 0.952 | 0.963 (0.619, 1.499) | 0.868 |
| GA | 41 (10.3%) | 40 (10.1%) |  |  |  |  |
| AA | 0 (0%) | 1 (0.3%) |  |  |  |  |
| MAF (A) | 0.06 | 0.05 |  |  |  |  |
| SNP9 (rs7647481) | | | | | | |
| GG | 365 (91.7%) | 350 (88.6%) | 0.703 (0.438, 1.128) | 0.144 | 0.716 (0.452, 1.135) | 0.155 |
| GA | 33 (8.3%) | 45 (11.4%) |  |  |  |  |
| AA | 0 (0%) | 0 (0%) |  |  |  |  |
| MAF (A) | 0.04 | 0.06 |  |  |  |  |
| SNP11 (rs2197423) | | | | | | |
| GG | 365 (91.7%) | 351 (88.9%) | 0.721 (0.449, 1.159) | 0.177 | 0.733 (0.462, 1.164) | 0.189 |
| GA | 33 (8.3%) | 44 (11.1%) |  |  |  |  |
| AA | 0 (0%) | 0 (0%) |  |  |  |  |
| MAF (A) | 0.04 | 0.06 |  |  |  |  |
| SNP14 (rs6802898) | | | | | | |
| CC | 365 (91.7%) | 351 (88.9%) | 0.721 (0.449, 1.159) | 0.177 | 0.733 (0.462, 1.164) | 0.189 |
| CT | 33 (8.3%) | 44 (11.1%) |  |  |  |  |
| TT | 0 (0%) | 0 (0%) |  |  |  |  |
| MAF (T) | 0.04 | 0.06 |  |  |  |  |

*MAF minor allele frequency; #Dominant Model mutation carriers vs. Non-mutation carriers; ^¶^Allele Model risk allele vs. wild type allele

**Supplementary Table 4** Association Genotype and allele frequencies of the 7 common variants with overweight

| Variants and genotypes | Overweight | Normal weight | OR (95% CI) | P value |
| --- | --- | --- | --- | --- |
| SNP1 (rs1801282) | | | | |
| Pro/Pro | 72(91.1%) | 558 (91.0%) | 0.987 (0.441, 2.207) | 0.975 |
| Pro/Ala | 7(8.9%) | 55 (9.0%) |  |  |
| Ala/Ala | 0 (0%) | 0 (0%) |  |  |
| SNP2 (rs12486170) | | | | |
| AA | 64 (81.0%) | 483 (78.8%) | 0.841 (0.480, 1.473) | 0.544 |
| GA | 15 (19.0%) | 124 (20.2%) |  |  |
| GG | 0 (0.8%) | 6 (1%) |  |  |
| SNP4 (rs7649970) | | | | |
| CC | 72 (91.1%) | 559 (91.2%) | 1.006 (0.450, 2.251) | 0.988 |
| CT | 7 (8.9%) | 54 (8.8%) |  |  |
| TT | 0 (0%) | 0 (0%) |  |  |
| SNP8 (rs17036333) | | | | |
| GG | 68 (86.1%) | 554 (90.4%) | 1.429 (0.735, 2.778) | 0.292 |
| GA | 11 (13.9%) | 57 (9.3%) |  |  |
| AA | 0 (0%) | 2 (0.3%) |  |  |
| SNP9 (rs7647481) | | | | |
| GG | 72 (91.1%) | 559 (91.2%) | 1.006 (0.450, 2.251) | 0.988 |
| GA | 7 (8.9%) | 54 (8.8%) |  |  |
| AA | 0 (0%) | 0 (0%) |  |  |
| SNP11 (rs2197423) | | | | |
| GG | 73 (91.7%) | 559 (91.2%) | 0.857 (0.362, 2.024) | 0.725 |
| GA | 6 (8.3%) | 54 (8.8%) |  |  |
| AA | 0 (0%) | 0 (0%) |  |  |
| SNP14 (rs6802898) | | | | |
| CC | 72 (91.7%) | 558 (91.0%) | 0.987 (0.441, 2.207) | 0.975 |
| CT | 7 (8.3%) | 55 (9.0%) |  |  |
| TT | 0 (0%) | 0 (0%) |  |  |

**Supplementary Table 5** Association Genotype and allele frequencies of the 7 common variants with overweight in GDM

| Variants and genotypes | Overweight | Normal weight | OR (95% CI) | P value |
| --- | --- | --- | --- | --- |
| SNP1 (rs1801282) | | | | |
| Pro/Pro | 51 (89.5%) | 265 (93.3%) | 1.605 (0.627, 4.112) | 0.324 |
| Pro/Ala | 6 (10.5%) | 19 (6.7%) |  |  |
| Ala/Ala | 0 (0%) | 0 (0%) |  |  |
| SNP2 (rs12486170) | | | | |
| AA | 46 (80.7%) | 218 (76.7%) | 0.772 (0.395, 1.510) | 0.450 |
| GA | 11 (19.3%) | 63 (22.2%) |  |  |
| GG | 0 (0.8%) | 3 (1.1%) |  |  |
| SNP4 (rs7649970) | | | | |
| CC | 51 (89.5%) | 266 (93.7%) | 1.698 (0.659, 4.375) | 0.273 |
| CT | 6 (10.5%) | 18 (6.3%) |  |  |
| TT | 0 (0%) | 0 (0%) |  |  |
| SNP8 (rs17036333) | | | | |
| GG | 51 (89.5%) | 255 (89.8%) | 1.033 (0.419, 2.547) | 0.945 |
| GA | 6 (10.5%) | 29 (10.2%) |  |  |
| AA | 0 (0%) | 0 (0%) |  |  |
| SNP9 (rs7647481) | | | | |
| GG | 51 (89.5%) | 265 (93.3%) | 1.605 (0.627, 4.112) | 0.324 |
| GA | 6 (10.5%) | 19 (6.7%) |  |  |
| AA | 0 (0%) | 0 (0%) |  |  |
| SNP11 (rs2197423) | | | | |
| GG | 51 (89.5%) | 265 (93.3%) | 1.605 (0.627, 4.112) | 0.324 |
| GA | 6 (10.5%) | 19 (6.7%) |  |  |
| AA | 0 (0%) | 0 (0%) |  |  |
| SNP14 (rs6802898) | | | | |
| CC | 51 (89.5%) | 265 (93.3%) | 1.605 (0.627, 4.112) | 0.324 |
| CT | 6 (10.5%) | 19 (6.7%) |  |  |
| TT | 0 (0%) | 0 (0%) |  |  |

**Supplementary Table 6** Comparison of demographic and clinical characteristics according to different genotypes

| **Clinical Profiles** | **CGGCC** | **TAATG** | **P value** |
| --- | --- | --- | --- |
| Number of subjects | 365 | 33 |  |
| Age(yr) | 31.31±4.32 | 32.33±4.66 | 0.196 |
| Mean delivery week (wk) | 38.45±5.58 | 38.24±1.84 | 0.829 |
| Pre-pregnancy BMI (kg/m^2^) | 21.45±3.46 | 21.25±3.37 | 0.780 |
| BMI prior to delivery (kg/m^2^) | 25.77±3.56 | 26.23±3.49 | 0.488 |
| Birth weight (g) | 3120.16±541.17 | 3185.76±555.32 | 0.506 |
| OGTT_0 h (mmol/L) | 4.61±0.75 | 4.76±0.82 | 0.288 |
| OGTT_1 h (mmol/L) | 9.85±1.55 | 9.78±1.70 | 0.815 |
| OGTT_2 h (mmol/L) | 9.00±1.45 | 9.22±1.30 | 0.417 |
| Cholesterol (mmol/L) | 5.23±1.27 | 5.26±0.86 | 0.910 |
| Triglyceride (mmol/L) | 1.95±0.98 | 1.92±1.15 | 0.894 |
| High-density lipoprotein (mmol/L) | 1.66±0.41 | 1.74±0.26 | 0.382 |
| Low-density lipoprotein (mmol/L) | 2.76±0.86 | 2.72±0.82 | 0.847 |
| HbA1C (%) | 5.20±0.49 | 5.15±0.33 | 0.595 |
| TSH (mIU/ml) | 1.46±1.11 | 1.52±0.97 | 0.777 |
| FT4 (pmol/l) | 14.51±2.86 | 14.40±3.95 | 0.417 |

BMI, body mass index; DM, diabetes mellitus; FSH, follicle-stimulating hormone; FT4, free thyroxine; GDM, gestational diabetes mellitus; wk, week; yr, years old.

**Legends for Supplementary tables**

**Supplementary table 1:** Primer sequences for promoter region and start codon of *PPARγ2* gene and Primer sequences for ChIP-PCR. 4 pairs of Primers were designed for promoter region and start codon of *PPARγ2* gene; 2 pairs of primers were designed for SNP3 for ChIP-PCR and 2 pairs of primers sequences for SNP12 and SNP13 for ChIP-PCR.

**Supplementary table 2:** Transcription factors siRNA sequences from genOFF^TM^ siRNA library.

**Supplementary Table 3:** In allele analysis, 7 common variants (rs1801282, rs12486170, rs7649970, rs17036333, rs7647481, rs2197423, and rs6802898) in the *PPARγ2* gene were genotyped. None were significantly associated with the risk of GDM.

**Supplementary Table 4:** Association 7 common variants (rs1801282, rs12486170, rs7649970, rs17036333, rs7647481, rs2197423, and rs6802898) with overweight. None were significantly associated with the risk of overweight in GDM and control.

**Supplementary Table 5:** Association 7 common variants (rs1801282, rs12486170, rs7649970, rs17036333, rs7647481, rs2197423, and rs6802898) with overweight in GDM. None were significantly associated with the risk of overweight in GDM.

**Supplementary Table 6:** There is no significance between T-A-A-T-G and C-G-G-C-C haplotype in demographic and clinical characteristics.
